# Association of serum klotho levels with different-staged vascular calcification status in patients with maintenance hemodialysis

**DOI:** 10.1186/s12882-022-02995-1

**Published:** 2022-11-19

**Authors:** Yan Lin, Jiayuan Huang, Meiyang Zhou, Cuiping Pan, Aiqin Shan, Canxin Zhou

**Affiliations:** Department of Nephrology, The Affiliated People’s Hospital, Ningbo University, Zhejiang 315040 Ningbo, China

**Keywords:** Vascular calcification, Maintenance hemodialysis, klotho, Association

## Abstract

**Background:**

Vascular calcification (VC) is suggested to be associated with serum klotho levels in patients with maintenance hemodialysis (MHD), whereas there is a lack of reports on the associations of VC status in whole arteries with serum klotho contents.

**Methods:**

One hundred forty eligible patients with MHD and a total of age-and gender-matched normal controls (NCs) were recruited. We analyzed the VC statuses of large arteries and peripheral muscular arteries by calculating the sum of scores from each artery. The levels of serum klotho were determined by ELISA. In addition, the relationship between serum klotho and VC status was evaluated using correlation analysis and regression analysis.

**Results:**

The VC severity in MHD patients tended to be worse in comparison with NCs. Serum klotho level in patients with MHD was lower than that in the NC subjects (​*P* < 0.0001), which was correlated with VC scores as reflected by correlation analysis and regression analysis. Serum klotho concentrations exhibited a dynamic decline along with increased VC status stages. Subjects with higher levels of serum klotho had a higher prevalence of cardiovascular events.

**Conclusion:**

Our study indicates serum klotho is strongly associated with VC status in a stage-dependent manner.

## Introduction

Cardiovascular disease (CVD) is the leading cause of mortality in patients with maintenance hemodialysis (MHD) [1]. Vascular calcification (VC) is an important pathology in CVD and is recognized as the predictor of CVD incidence and mortality [2]. VC is a multifactor-mediated, progressive and complicated process, remaining a challenge to elucidate the underlying mechanisms. Monitoring the process of VC represents the potential of intervening the VC-associated CVD, especially for MHD patients. Currently, the diagnosis of VC in large arteries, such as the abdominal aorta, by imaging examination reaches a high precision [3]. However, imaging examinations are not sufficient to uncover VC in the small artery. Thus, screening reliable biomarkers that predict VC in extensive arteries is highly imperative.

Klotho is identified as an anti-aging agent, which might act as a protective role in the development of VC [4]. The loss-off function of the klotho gene contributes to premature syndrome in mice, including VC, infertility, arteriosclerosis, and skin atrophy [5]. In addition, an extrinsic supplement of soluble klotho mitigates VC in vivo [3] and in vitro [6]. Importantly, serum klotho levels are related to the risk of severe abdominal aortic calcification and can be treated as a predictor of aortic calcification in the abdominal artery [7]. However, these studies only focus on the role of serum klotho in the VC of large arteries. Still, the comprehensive associations of serum klotho levels with VC status of the whole body as well as predictive values are not well documented. In addition, the changes in serum klotho levels at different stages of VC are poorly understood.

Given the possibility of klotho in predicting VC in large vessels, the objective of the present study is to investigate the relationship between VC status of the whole body and serum klotho levels in MHD patients with different stages of VC.

## Methods and materials

### Subjects and data acquisition

140 MHD patients as well as an equal number of normal controls (NCs) were consecutively recruited from our hospital. MHD patients were included if they meet the following criteria: (1) having dialysis already for 4 months with a routine dialysis schedule; (2) absence of serious infections or autoimmune diseases; (3) free from cancers. Conversely, patients were not eligible if they have at least one following criteria: malignancy, infection, and impaired liver function. Clinical data of all participants, including age, sex, hypertension, diabetes, cardiovascular events, drug history, etc. were collected. The cardiovascular events include stroke, non-fatal myocardial infarction, and revascularization [8]. Drug history was defined as taking a renin-angiotensin system (RAS) inhibitor or an angiotensin receptor inhibitor (ARB) within three months. Malnutrition was evaluated using the phenotypic criteria (weight loss, low body mass index, and reduced muscle mass) according to a previous study [9]. In addition, we obtained written informed consent from all participants enrolled.

### Blood sampling and measurement of serum klotho

We collected fasting blood from all participants between 07:00 and 09:00 to reduce potential bias before dialysis. After being centrifuged at 3,000 rpm for 15 min at room temperature, serum was transferred into a new tub and stored at -80℃ until the determination. We determined the serum klotho concentrations using a commercially available ELISA according to the manufacturer (R&D Systems). Briefly, 100 µL of serum was added into the recommended microplate with a cover for 2 h-incubation at room temperature. After being washed, the detection antibody was added for another 2 h-incubation at room temperature. Finally, 100 µL of substrate solution was added to develop the positive signals and then 50 µL of stop solution was used to stop the reaction. The concentration was determined by a microplate reader.

### VC status evaluation

VC statuses of large arteries and peripheral muscular arteries in MHD patients and NCs were evaluated according to a previous study [10]. Firstly, plain radiographic images of the lateral abdomen, pelvis, and hands were collected. Then, the abdominal aortic artery, iliac arteries, femoral arteries, radial arteries, and digital arteries were comprehensively examined by two professional radiologists who are blinded to the participants to determine the presence of VC in each artery. The presence of calcification in each artery is termed as 1 and absence as 0. The VC status is defined as the sum of scores from each artery. In addition, we classified the VC status into different stages according to the total scores. The classifying criteria are as follows: 1–3 as mild calcification, 4–6 as moderate calcification, and over 7 as severe calcification.

### Statistical analysis

All data presented in the study were analyzed by SPSS 22.0 (USA) and graphed prism 9.0. If continuous data comply with normal distribution, they are presented as mean ± SD and examined by independent t-tests. Comparison of categorical data between two groups was examined by chi-square tests or rank sum tests. Pearson correlation analysis or Spearman correlation analysis was applied to determine the association between VC status and serum klotho levels. T Differences among multiple groups were analyzed by one-way ANOVA following Turkey’s post hoc analysis. The linear regression model was utilized to determine the association between VC scores and serum klotho levels in MHD patients. The logistic regression analysis was conducted to analyze the relationship between serum klotho levels and cardiovascular events. A *P* value < 0.05 was considered a significant difference.

## Results

### Characteristics of the study participants

The demographic characteristics of the study participants were presented in Table 1. There were no differences in age, gender, blood pressure, hypertension, and diabetes (Table 1, *P* > 0.05). Patients with MHD had higher levels of triglyceride and cholesterol than those in NCs (Table 1, *P* < 0.001). Additionally, higher levels of serum creatine and phosphorus as well as lower levels of serum albumin and high-density lipoprotein were observed (Table 1, *P* < 0.001), indicating an impaired kidney function. Patients with MHD had higher levels of parathyroid hormone (PTH) than those in NCs (Table 1, *P* < 0.001).

**Table 1:** Demographic characteristics

Variable	NCs	MHD patients	Statistics	*P*-value
No. of patients	140	140		
Age (years)	51.13 ± 7.4	52.15 ± 8.8	t = 1.050	0.295
Male (%)	50 (35.7)	53 (53)	χ^2^ = 37.9	0.710
Systolic pressure (mmHg)	137.2 ± 16.1	139.2 ± 15.3	t = 1.066	0.288
Diastolic pressure (mmHg)	82.3 ± 8.2	83.3 ± 7.4	t = 1.071	0.285
Triglyceride (mmol/L)	1.34 ± 0.65	1.57 ± 1.06	t = 2.189	0.003
Cholesterol (mmol/L)	3.21 ± 0.42	3.60 ± 0.82	t = 5.008	< 0.001
Serum creatinine (µmol/L)	176.5 ± 86.2	734.4 ± 150.17	t = 38.123	< 0.001
Serum albumin (g/L)	43.8 ± 5.16	35.3 ± 4.20	t = 15.116	< 0.001
Hypertension (%)	39 (27.9)	43 (30.7)	χ^2^ = 0.276	0.599
Diabetes (%)	37 (26.4)	43 (30.7)	χ^2^ = 0.630	0.427
Dialysis duration (years)	NA	6.30 ± 1.52	NA	NA
Malnutrition	5 (3.6)	23 (16.4)	χ^2^ = 12.86	< 0.001
Vitamin D (ng/mL)	24.2 ± 5.20	25.2 ± 6.5	t = 1.42	0.156
Calcium (mmol/L)	2.35 ± 0.52	1.99 ± 0.12	t = 7.982	< 0.001
PTH (pg/mL)	56.2 ± 13.20	244 ± 63.20	t = 34.42	< 0.001
Drug history (%)	35 (25)	84 (74.3)	χ^2^ = 35.09	< 0.001
Serum phosphorus (mmol/L)	1.20 ± 0.32	1.80 ± 0.62	t = 10.175	< 0.001
High-density lipoprotein (mmol/L)	1.49 ± 0.42	1.01 ± 0.29	t = 11.13	< 0.001
Low-density lipoprotein (mmol/L)	2.71 ± 0.55	3.21 ± 0.39	t = 8.774	< 0.001

*NA* Indicates not applicable, *PTH* Parathyroid hormone

### Comparison of VC status between NCs and MHD patients

The frequency of VC in NCs was remarkably lower than that in MHD patients (15% vs. 70%; χ^2^ = 86.65, *P* < 0.01). In addition, the distribution of VC severity in MHD patients differed from that in NCs (Z = 9.489, *P* < 0.01, Table 2). The VC severity in MHD patients tended to be worse in comparison with NCs..

**Table 2:** Comparison of VC severity between NCs and MHD patients

Groups	Non-VC	Mild VC	Moderate VC	Severe VC
NCs	119 (85.0%)	12 (8.57%)	5 (3.57%)	4 (2.86%)
MHD patients	42 (30.0%)	25 (17.86%)	32 (22.86%)	41 (29.29%)
Z value	9.489 < 0.001			
*P*-value				

### Comparison of serum klotho levels between NCs and MHD patients

**Fig. 1:**
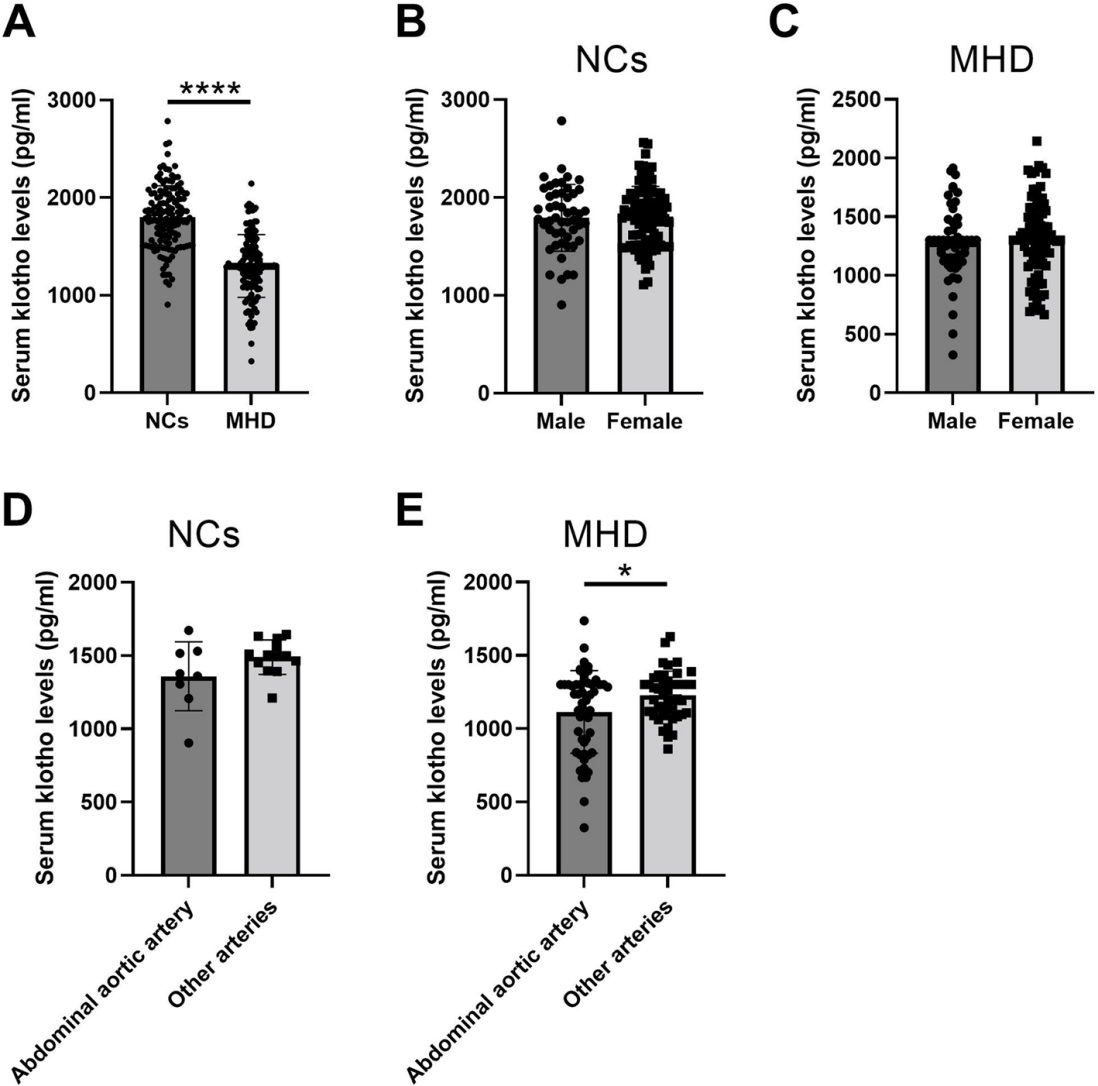
Comparison of serum klotho levels between two groups. **A** Comparison of serum klotho concentrations between normal controls and MHD patients. **B**, Comparison of serum klotho concentrations between males and females in normal controls. **C**, Comparison of serum klotho concentrations between males and females in MHD patients. **D**, Comparison of serum klotho concentrations between subjects with VC on abdominal aortic artery or other types of arteries in normal controls. **E**, Comparison of serum klotho concentrations between subjects with VC on abdominal aortic artery or other types of arteries in MHD patients. **** indicates *P* < 0.0001, * indicates *P* < 0.05. *N* = 140 per group for Fig. 1A; *N* = 50 for the male group and 90 for the female group in Fig. 1B; *N* = 53 for the male group and 87 for the female group in Fig. 1C; *N* = 8 for group of abdominal aortic artery and 13 for group of other arteries in Fig. 1D; *N* = 54 for group of abdominal aortic artery and 44 for group of other arteries in Fig. 1E. The error bars present the SD

First, to interrogate the changes in serum klotho concentrations, we examined serum klotho levels using ELISA. Serum klotho levels in patients with MHD were lower than those in the NC subjects (​*P* < 0.0001, Fig. 1 A). Sub-analysis revealed that there were no significant differences in klotho contents between males and females in the two groups (Fig. 1B and C, *P* > 0.05). In addition, we also compared the difference in serum klotho levels among subjects with VC in abdominal aortic arteries or other types of arteries. We found higher levels of serum klotho in subjects with VC on other types of arteries than that in subjects with VC on an abdominal aortic artery in the MHD group (Fig. 1E, *P* < 0.05), but not in NCs (Fig. 1D, *P* > 0.05).

### Correlation of serum klotho levels with VC scores in NCs and MHD patients

**Fig. 2:**
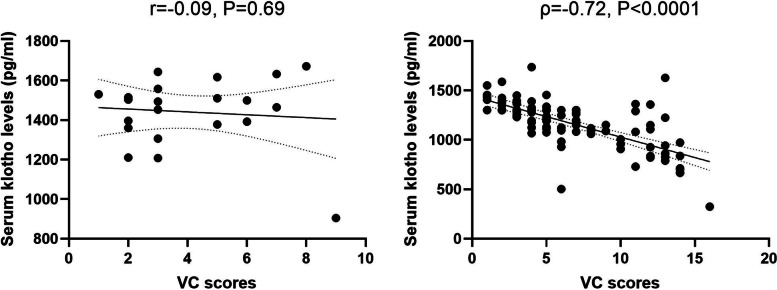
Association of serum klotho levels with VC scores. Correlation of serum klotho concentrations with VC scores in VC-positive normal controls (left). B, Correlation of serum klotho concentrations with VC scores in VC-positive MHD patients. *N* = 21 for NCs and *N* = 98 for VC scores. The error bars present the SD

We conducted a Pearson correlation analysis to examine whether serum klotho levels are correlated with creatinine VC scores. Unfortunately, we found there was a lack of significant correlation between serum klotho levels with VC scores in NCs (Fig. 2, *P* > 0.05), whereas a profound association between serum klotho levels with VC scores was observed in MHD patients (Fig. 2, *P* < 0.0001). In addition, the linear regression models were utilized to determine whether serum klotho levels are correlated with VC scores under the condition of other confounders in patients with MHD. The data showed serum klotho levels were indeed correlated with VC scores with a beta value of -0.559 (Table 3).

**Table 3:** Linear regression models to evaluate the relationship between serum klotho levels and VC scores in patients with MHD

Variables	Unadjusted		Adjusted	*P* value
	B	SD	β	
Serum klotho levels	-0.008	0.001	-0.555	< 0.001
Age	0.064	0.027	0.123	0.018
Diabetes	1.331	0.490	0.134	0.008
Malnutrition	1.866	0.614	0.152	0.003
Serum phosphorus	0.917	0.356	0.123	0.011
Constant	3.450	2.646		0.195

### Comparison of serum klotho levels at different VC status stages in MHD patients

**Fig. 3:**
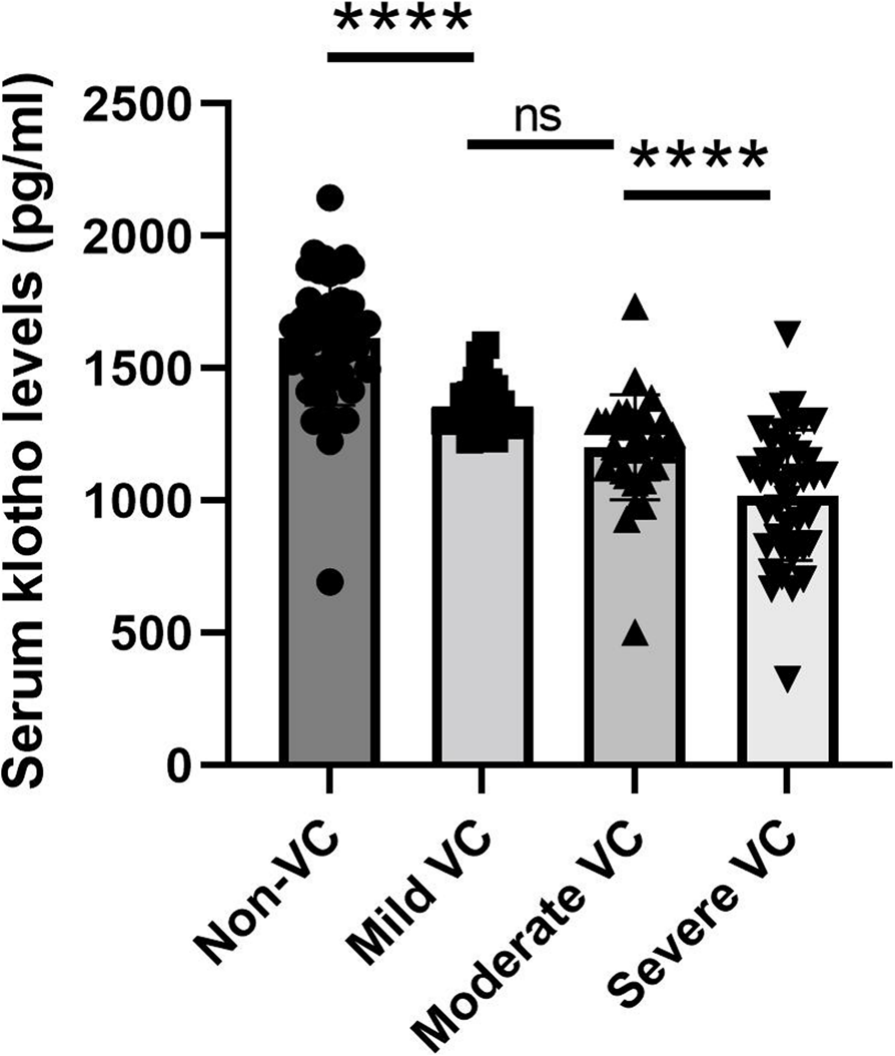
The serum klotho levels in MHD patients at different-staged VC status. Comparison of serum klotho levels in Correlation of serum klotho concentrations at different-staged VC status in MHD patients. *N* = 42 for non-VC, *N* = 25 for mild VC, *N* = 32 for moderate VC, and *N* = 41 for severe VC. The error bars present the SD

Importantly, we found serum klotho concentrations exhibited a dynamic decline along with increased VC status stages (Fig. 3). Patients with severe VC had the lowest serum klotho levels, while non-VC patients had the highest serum klotho concentrations in the MHD group.

### The relationship between serum klotho levels and cardiovascular events

To further uncover the relationship between serum klotho levels and cardiovascular events, we defined the high levels of serum klotho and low levels of serum klotho according to the cutoff value of serum klotho levels in different cohorts. Then, we compared the prevalence of cardiovascular events between the high-level group and the low-level group. Among all cases, the high-level group had a lower prevalence rate of cardiovascular events than the low-level group (10% vs. 25%, *P* < 0.05), whereas the prevalence rate of cardiovascular events did not differ between the high-level group and low-level group in NCs (*P* > 0.05). In addition, compared with the high-level group, a higher prevalence rate of cardiovascular events in the low-level group was observed in MHD patients (24% vs. 35%, *P* < 0.05). Furthermore, we conducted the multivariable logistic regression to furtherly assess the association of serum klotho with clinical outcomes. The results showed that serum klotho is a potentially protective factor for cardiovascular events [Odds ratio (OR) = 0.883, *P* < 0.001].

## Discussion

In the present study, we aimed to investigate the levels of serum klotho levels in MHD patients with different-staged VC as well as its association with VC status. We found the serum klotho concentration was diminished and presented a progressive decline along with the VC severity in MHD patients. Besides, the total VC scores were negatively correlated with serum klotho levels in MND patients but not in NCs. Lastly, we found a satisfying diagnostic value of serum klotho levels for VC status.

Klotho is presented in two forms, soluble one and membrane-anchored one, and both are prominently produced in the brain and kidney. Membrane-anchored klotho always exerts its functions as co-receptors by FGF-23 in a specific high-affinity form [11]. In contrast, soluble klotho can broadly function as a paracrine factor or a hormone to illicit downstream actions, such as anti-aging process, thereby conferring the potential of functioning as a biomarker to reflect the physiological or pathological process in organs that are its targets or sources to some extent. Indeed, previous studies found the declined levels of serum klotho are associated with advances in chronic kidney disease (CKD) and cardiovascular diseases [10, 12, 13].

The epidemiologic study has well documented that MHD patients are concomitant with VC [14]. Our study revealed the frequency of VC in MHD is 70%, which was profoundly higher than that in NCs. VC is characterized by the active deposition of calcium phosphate on the vascular walls, which leads to the high mortality of MHD. Several studies indicated the association of serum klotho levels are correlated with VC in large vessels in MHD patients, such as abdominal aortic calcification [7, 15]. However, these studies only focus on the large vessels but do not reveal the associations of VC status in whole arteries with serum klotho contents. We postulate that incorporating arteries as more as possible to evaluate the VC status of the whole body would better reflect its association with serum klotho. In this regard, we evaluated the VC status by calculating the VC score from the abdominal aortic artery, iliac arteries, femoral arteries, radial arteries, and digital arteries, and found the VC status of whole arteries was strongly associated with serum klotho concentrations in MHD patients but not in NCs. The discrepancy of associations might be attributed to the dysfunction of kidneys of MHD patients but not NCs, as the kidney is the principal source of peripheral klotho [16]. In addition, to our knowledge, there is a lack of comprehensive evidence regarding the changes of serum klotho in different stages of VC in MHD patients. Importantly, our study for the first time found serum klotho levels decline along with the advance of VC status in large and peripheral muscular vessels. Taken together, our study provides additional evidence that serum klotho concentrations are strongly associated with the VC status of the whole body in MHD patients.

It is intriguing to postulate the mechanism underlying the association of serum klotho with VC. VC is characterized by the trans-differentiation of vascular smooth muscle cells (VSMCs) into osteogenic-like cells [17]. CKD-induced mineral bone disorder syndrome is a driver for the trans-differentiation of VSMCs. Accumulated evidence has well demonstrated that hyperphosphatemia is common in MHD and is a contributor to VC [18, 19]. Indeed, our results also found a higher level of serum phosphate and lower klotho level in patients with MHD. Importantly, klotho deficiency induces a higher serum phosphate level and more severe VC than that in CKD mice [20]. Conversely, enhancing the levels of klotho by genetic ways or supplementation could rescue the hyperphosphatemia and severity of VC [20]. In MHD patients, a lower level of klotho may accelerate the formation of VC by regulating the homeostasis of phosphorus metabolism, thereby showing an association of serum klotho with VC.

There are some limitations in our study. First, this was a cross-sectional study and only explored associations between serum klotho levels and VC scores, which cannot refer to the causality. Further studies are required to determine the causality between serum klotho levels and VC status. Second, the sample of our cohort was relatively small, bigger cohorts are required to validate our results. Third, we cannot rule out the residual confounding from unmeasured or unknown variables.

In conclusion, our study indicates serum klotho is strongly associated with VC status in a stage-dependent manner. In addition, the level of serum klotho is a reliable predictor for VC.

## Data Availability

All data supporting the conclusions of this article are included within the article.

## References

[1] Wang M, Wang M, Gan LY, Li SJ, Hong N, Zhang M (2009). Vascular calcification in maintenance hemodialysis patients. Blood Purif.

[2] Zeng C, Guo C, Cai J, Tang C, Dong Z (2018). Serum sclerostin in vascular calcification and clinical outcome in chronic kidney disease. Diabetes Vasc Dis Res.

[3] Hum JM, O’Bryan LM, Tatiparthi AK, Cass TA, Clinkenbeard EL, Cramer MS, Bhaskaran M, Johnson RL, Wilson JM, Smith RC (2017). Chronic Hyperphosphatemia and Vascular Calcification Are Reduced by Stable Delivery of Soluble Klotho. J Am Soc Nephrol.

[4] Yu L, Li M (2020). Roles of klotho and stem cells in mediating vascular calcification (Review). Experimental and therapeutic medicine.

[5] Cardoso AL, Fernandes A, Aguilar-Pimentel JA, de Angelis MH, Guedes JR, Brito MA, Ortolano S, Pani G, Athanasopoulou S, Gonos ES (2018). Towards frailty biomarkers: Candidates from genes and pathways regulated in aging and age-related diseases. Ageing Res Rev.

[6] Chen T, Mao H, Chen C, Wu L, Wang N, Zhao X, Qian J, Xing C (2015). The Role and Mechanism of α-Klotho in the Calcification of Rat Aortic Vascular Smooth Muscle Cells. Biomed Res Int.

[7] Wang F, Zheng J (2022). Association between serum alpha-Klotho and severe abdominal aortic calcification among civilians in the United States. Nutr metabolism Cardiovasc diseases: NMCD.

[8] Jankowich M, Choudhary G (2020). Endothelin-1 levels and cardiovascular events. Trends Cardiovasc Med.

[9] Corish CA, Bardon LA (2019). Malnutrition in older adults: screening and determinants. Proc Nutr Soc.

[10] Adragao T, Pires A, Lucas C, Birne R, Magalhaes L, Gonçalves M, Negrao AP (2004). A simple vascular calcification score predicts cardiovascular risk in haemodialysis patients. Nephrol Dial Transplant.

[11] Dalton GD, Xie J, An SW, Huang CL (2017). New Insights into the Mechanism of Action of Soluble Klotho. Front Endocrinol (Lausanne).

[12] Cai H, Zhu X, Lu J, Zhu M, Liu S, Zhan Y, Ni Z, Gu L, Zhang W, Mou S (2021). A Decreased Level of Soluble Klotho Can Predict Cardiovascular Death in No or Mild Abdominal Aortic Calcification Hemodialysis Patients. Front Med.

[13] Neyra JA, Hu MC, Moe OW (2020). Klotho in Clinical Nephrology: Diagnostic and Therapeutic Implications. Clin J Am Soc Nephrology: CJASN.

[14] Martins MTS, Matos CM, Lopes MB, Kraychete AC, Lopes GB, Martins MTS, Fernandes JL, Amoedo MK, Neves CL, Lopes AA (2021). Vascular calcification by conventional X-ray and mortality in a cohort of predominantly African descent hemodialysis patients. Int J Artif Organs.

[15] Milovanova LY, Lysenko Kozlovskaya LV, Milovanova SY, Taranova MV, Kozlov VV, Reshetnikov VA, Lebedeva MV, Androsova TV, Zubacheva DO, Chebotareva NV (2020). [Low serum Klotho level as a predictor of calcification of the heart and blood vessels in patients with CKD stages 2-5D]. Ter Arkh.

[16] Lindberg K, Amin R, Moe OW, Hu MC, Erben RG, Ostman Wernerson A, Lanske B, Olauson H, Larsson TE (2014). The kidney is the principal organ mediating klotho effects. J Am Soc Nephrol.

[17] Ray M, Jovanovich A (2019). Mineral Bone Abnormalities and Vascular Calcifications. Adv Chronic Kidney Dis.

[18] Ritter CS, Slatopolsky E (2016). Phosphate Toxicity in CKD: The Killer among Us. Clin J Am Soc Nephrology: CJASN.

[19] Yamada S, Giachelli CM (2017). Vascular calcification in CKD-MBD: Roles for phosphate, FGF23, and Klotho. Bone.

[20] Hu MC, Shi M, Zhang J, Quiñones H, Griffith C, Kuro-o M, Moe OW (2011). Klotho deficiency causes vascular calcification in chronic kidney disease. J Am Soc Nephrol.

